# Deep learning based attention enhanced phylogenetic radial basis function networks (AE-PRBFN) for genomic codon usage classification across species

**DOI:** 10.1038/s41598-026-48503-5

**Published:** 2026-06-29

**Authors:** Yaseein Soubhi Hussein, Aitizaz Ali, Anjum Shahzad, Tahir Mehmood, Muhammad Ilyas, Mohamed Shabbir Abdulnabi

**Affiliations:** 1 Department of Information Systems and Computer Science Ahmed Bin Mohammed Military College (ABMMC), P.O. Box: 22988, Doha, Qatar; 2 Strategic Research Institute, Asia Pacific University of Technology and Innovation (APU), Kuala Lumpur, 57000 Malaysia; 3 School of Natural Sciences (SNS), National University of Sciences and Technology (NUST), Islamabad, Pakistan; 4 Kohsar University Murree, Murree, Punjab Pakistan; 5 School of Technology (SOT), Asia Pacific University of Technology and Innovation(APU), Kuala Lumpur, 57000 Malaysia

**Keywords:** Radial Basis Function Neural Network, Phylogenetic, Attention Enhanced, Species, Genomic, Evolutionary, Computational biology and bioinformatics, Genetics, Plant sciences

## Abstract

Understanding codon usage patterns is essential for genomic classification and offers insights into the evolutionary and functional characteristics of species. This study focuses on classifying four agriculturally significant crop species Triticum aestivum (wheat), Oryza sativa (rice), Hordeum vulgare (barley), and Brachypodium distachyon using their codon usage biases. Traditional methods often struggle with high-dimensional genomic data, prompting the need for advanced deep learning techniques. Here, we introduce Attention Enhanced Phylogenetic Radial Basis Function Networks (AE-PRBFN), a novel neural architecture designed to improve classification accuracy and efficiency. We analyzed 253,076 high-quality coding sequences (131,656 wheat; 31,970 rice; 37,932 barley; 51,518 Brachypodium) using absolute codon frequencies (64 features) as input. AE-PRBFN achieved 100% accuracy, significantly outperforming standard RBFN, Multilayer Perceptron, Support Vector Machines, and Random Forest. Moreover, AE-PRBFN required only 2 training epochs (10-fold cross-validation) versus approximately 200 epochs for standard RBFN, demonstrating substantially faster convergence. AE-PRBFN captures subtle, species-specific codon usage signatures that distinguish these four Poaceae crops with perfect accuracy, establishing that absolute codon frequency encodes sufficient phylogenetic signal for taxonomic classification.

## Introduction

Codon usage patterns analysis gives some fundamental facts about evolutionary biology, regulation of gene expression, and species-specific signature genome^1^. The non-random use of codons encoding identical amino acid is referred to as synonymous codon bias, which differs among organisms because of interplays among mutational, natural selection, and translational optimization factors^2^. This is of special importance when applied to plant genomics where codon preferences can be used to improve crops and design transgenes^3^. Economically important grasses, such as Triticum aestivum (wheat), Oryza sativa (rice), Hordeum vulgare (barley) and the model plant Brachypodium distachyon, have unique genomic architecture, and such architecture is a result of unique evolutionary pathways^4^. However, more complex analytical solutions are usually required to extract the complex, high degree of relationships inherent in codon frequency data which are not usually represented in conventional bioinformatics tools.

The recent field of machine learning (ML) has transformed the area of genomic research because it allows analyzing vast biological samples^5^. Different ML and deep learning (DL) strategies have been used in codon usage bias (CUB) research. Conventional methods involve Support Vector Machines (SVMs) and Random Forests which have been applied to the classification of species in terms of codon frequencies^6^. Most recently, Convolutional Neural Networks (CNNs) and Recurrent Neural Networks (RNNs) have been implemented as deep learning frameworks to learn spatial and temporal correlations in genomic data, they have been shown to be more successful in capturing complex patterns^7,8^. Especially attention mechanisms have proved to be an effective instrument in bioinformatics. Attention has enhanced activities such as predicting genes, detecting regulatory elements and predicting protein structures by permitting models to dynamically adjust the relative importance of various input features or the positions of the sequence during prediction^9,10^. As an example, frameworks such as multidimensional cooperative analysis (MCA) to disease diagnosis or light weight dual stream learning (LDSL)s to plant disease detection make use of grouped or structured attention strategy, allowing models to pay attention to the most informative genomic regions^11,12^. ML models have also been given evolutionary or phylogenetic inputs to enhance their biological significance. Comparative genomics has studied phylogeny-aware techniques, like the regularization with evolutionary distances or phylogeny tree^13^. Yet, there is a significant gap: no existing research has included phylogenetic constraints right into the basis structure of a Radial Basis Function Network (RBFN) through the evolutionally informed centroid initialization and a refinement process of attention-based feature refinement, aimed specifically at codon usage data^14^^[,15^.

Radial Basis Function Networks (RBFNs) are conspicuous to depicting non-linear relationships of complex nature by localized kernel transformations^16^. Traditional RBFNs map input data into a higher dimensional space with functions that are based on known locations^17^. These methods, however, have drawbacks: they use centroid points in a static way (usually based on generic clustering, such as the K-means algorithm), they are sensitive to high-dimensional noise, and they do not work optimally when the phylogenetic-ally related species contain overlapping codon patterns^18^. These limitations decrease the accuracy of classification as well as raise computational intensity of the genomic information which have minor inter-species differences^19^. Although the recent DL models can be highly accurate, they tend to be black box and they can be only partially interpretable about the evolutionary forces behind the observed codon bias patterns^20^.

To resolve these issues, we present an Attention-Enhanced Phylogenetic Radial Basis Function Network (AE-PRBFN) using Codon Usage-Defined Centroids. The three important innovations incorporated in this new framework to specifically develop codon-based genomic classification are:

1. Phylogenetically Informed Centroids: We introduce a technique in which centroid values of RBFNs are not randomly chosen or randomly chosen based on general clustering but are actually directly obtained as a result of the evolutionary relationships of the target organisms (wheat, rice, barley, Brachypodium). This biologically prior knowledge is encoded as part of the model-learning context, and this influences the model to learn in a phylogenetic-ally constrained feature space.
2. Attention-Enhanced Feature Refinement: An attention system balances high-dimensional codon frequency data (64 dimensions) by selectively weighting features with their species specific discriminatory power^21^. This paper is inspired by structure attention in bioinformatics^11,12^ but this time unites it in order to optimize codon characteristics to an RBFN.
3. Integrated Probabilistic Optimization: The model is dynamic, centroid positions are adjusted by the training process, enabling the phylogenetic-ally initialized centroid to be optimized by the data^22^.

This architectural phenomenon is a break with the current practices. It does not just look at phylogeny as an input or a tool of post-hoc analysis but makes it structural to the learning process. This paper involves the construction and testing of the AE-PRBFN model based on absolute codon frequencies obtained as a result of the coding sequences (CDS) of the four grass species. We show its behavior in contrast to standard RBFN and other current ML and DL baselines, a new paradigm of integration of biological knowledge into machine learning architectures of genomic analysis has been set.

## Methods

### Data preparation

In February 2025, the coding sequences of the entire genomes of Triticum aestivum (Wheat), Oryza sativa (Rice), Hordeum vulgare (Barley), and Brachypodium distachyon (BD) were obtained in FASTA format from the EnsemblPlants database. The raw sequence data supporting the findings of this study are publicly available from the Ensembl Plants database (http://plants.ensembl.org/). The fully processed dataset (codon frequency features and labels) and all codes required to reproduce it including data retrieval, filtering, and feature extraction are publicly available on GitHub at https://github.com/anjumstat/AE_PRBFN_Deep_Learning_Classification.git.

### Classification performance evaluation metrics

The standard classification metrics such as accuracy, precision, recall, F1-score and Matthews Correlation Coefficient (MCC)^23–28^-^29^ were used to measure model performance. Accuracy is the rate of the properly determined instances$\:(TP+TN/TP+TN+FP+FN)$, here TP stands for true positive TN for true negatives, FP for false positives and FN for false negatives. Although simple, accuracy is misguided in unbalanced genomic data but our four-species codon frequency task is balanced and accuracy is informative^23,24^. Precision $\:(TP/TP+FP)$ measures the accuracy of positive predictions and this is important in that the genes that are described as belonging to a particular species are biological^25,26^. Recall ($\:TP/TP+FN)$ is the overlap of all the appropriate instances that the model will observe, such as those rare codon usage patterns^25,26^. F1-score (harmonic mean of both precision and recall) is the measure that halts a balance between these indicators and is suitable in case of imbalanced datasets, where models harming the performance of the minority classes are penalized^24^. MCC (range − 1 to + 1) uses all the confusions matrix $\:\left(TP*TN-FP*FN/\sqrt{(TP+FP)(TP+FN)(TN+FP)(TN+FN)}\right)$ and gives a sound performance evaluation in fluctuating amounts of classes. In the research, MCC demonstrated enhanced classifications that were not reflected by accuracy or F1-score only^27,28^. Together, these measures indicated that AE-PRBFN is able to classify codon usage with high-precision in all four species and prevent over-fitting^5,29^.

### Data splitting & 10 fold cross validation

The dataset was divided into two portions: 10% was taken as the test set, while the remaining 90% was taken as training data for training the deep learning models^30^. The test data was kept entirely independent and solely reserved for the final evaluation of the models. The training data was split into 10 nearly equal segments, known as folds. In each iteration, one fold served as the validation set while the remaining nine were used for training. This process was repeated 10 times, with a different fold was taken as hold out each time, generating 10 distinct accuracy estimates. For instance, the model’s performance was measured individually for each validation fold, such as Accuracy$\:{}_{1}$, Accuracy$\:{}_{2}$,., Accuracy$\:{}_{10}$. The overall cross-validation accuracy was computed as the mean across all 10 folds, using the formula below:-

$$\:{\mathrm{CV}}_{K}=\frac{1}{10}\sum\:_{i=1}^{10}{\mathrm{Accuracy}}_{i}$$.

The same method was used to compute training accuracy. The “Classification Performance Evaluation Matrices” were computed on the basis of the test data. The following three steps explicitly explains the model training procedure:-.

Step 1: A 90 − 10% partition to create a held-out test set^31^^[,32^.

Step 2: 10-fold cross-validation performed solely on the 90% training set.

Step 3: Final evaluation of the best model from each fold on the blind 10% test set.

In genomic research, this approach is commonly used to evaluate deep learning models in applications like variant prioritization and gene expression forecasting, where small datasets increase the risk of over fitting. For instance, investigations assessing variant classification systems often employ 10-fold cross-validation to maintain consistent accuracy across different genomic segments^19^. Its ability to balance computational efficiency with statistical robustness has established it as the preferred benchmark method in genomic tools^5^.

### Standard radial basis function neural network (RBFN)

The conventional RBFN architecture^33^ maps input vectors x ∈ ℝᵈ to outputs via a nonlinear transformation using radial basis functions (RBFs). The network comprises three layers:

4. (1) Input Layer: Receives feature vector x (e.g., codon frequencies)^34^^[,35^.
5. (2) Hidden Layer: Computes activations using Gaussian RBFs centered at prototypes $\:{\mu\:}_{j}\in\:{\mathbb{R}}^{d}$.

1 $$\:{\varphi\:}_{j}\left(x\right)=\mathrm{e}\mathrm{x}\mathrm{p}\left(-\frac{2}{{\sigma\:}_{j}^{2}}\parallel\:x-{\mu\:}_{j}{\parallel\:}^{2}\right),\:j=1,\dots\:,k$$

Where $\:{\sigma\:}_{j}$ controls the width of the j-th RBF, and k is the number of centroids typically determined by K-means clustering^21^.Output Layer: Produces predictions through a linear combination^36^:

2 $$\:f\left(x\right)=\sum\:_{j=1}^{k}{w}_{j}\hspace{0.17em}{\varphi\:}_{j}\left(x\right)+b$$

Where $\:{\prime\:}{w}_{j}{\prime\:}$trainable weights and ‘b’ are is the bias term. For classification, a softmax function normalizes outputs to probabilities^37^:

3 $$\:p(y=c\mid\:x)=\frac{\mathrm{e}\mathrm{x}\mathrm{p}\left({f}_{c}\right(x\left)\right)}{\sum\:_{i=1}^{C}\mathrm{e}\mathrm{x}\mathrm{p}\left({f}_{i}\right(x\left)\right)}$$

With C classes. Training involves two stages:

(1) Unsupervised centroid initialization via K-means, and^38^^[,39^^[,40^.

(2) Supervised weight optimization using least squares^41^. Unlike the proposed hybrid AE -PRBFN, classical implementations fix centroids and widths ($\:{\sigma\:}_{j}$) after initialization. The model minimizes cross-entropy loss via Adam optimization^42^.

4 $$\:L=-\sum\:_{i=1}^{N}\sum\:_{c=1}^{C}{y}_{ic}\mathrm{l}\mathrm{o}\mathrm{g}p(y=c\mid\:{x}_{i})$$

Where $\:{y}_{ic}$ represents one-hot encoded labels.

### Proposed AE PRBFN

The proposed **AE-PRBFN** integrates Radial Basis Function Networks (RBFNs)^16^ with attention mechanisms^43^ and codon usage defined constraints^44^ to model species-specific codon usage patterns, this provides an implicit data driven constraint. Below, we formalize its key components mathematically^45^.

### Input representation and preprocessing

Let $\:X\in\:{\mathbb{R}}^{N\times\:64}$ denote the matrix of codon absolute frequencies for $\:N$ samples, where each row $\:{x}_{i}$ represents the codon absolute frequencies of 64 codons for the $\:i$-th sample. The species labels $\:{y}_{i}\in\:\{1,2,3,4\}$ are one-hot encoded in to$\:Y\in\:\{0,1{\}}^{N\times\:4}$. The input is standardized^46^ as:

5 $$\:{X}_{\mathrm{std}}=\frac{X-\mu\:}{\sigma\:},$$

Where $\:\mu\:\:\mathrm{a}\mathrm{n}\mathrm{d}\:\sigma\:$ represents the feature-wise means and standard deviations^47,]^^48^.

### Codon usage defined RBF centroids

The centroids $\:C\in\:{\mathbb{R}}^{K\times\:64}$ (where $\:K$ is the number of centroids) are initialized using K-means++^49^ on $\:{X}_{\mathrm{std}}$, with a codon usage bias: samples from the same species are prioritized during centroid^50^ assignment. The centroid update rule is:

6 $$\:{c}_{k}=\frac{1}{\left|{S}_{k}\right|}\sum\:_{i\in\:{S}_{k}}{x}_{i},$$

Where $\:{S}_{k}$ is the set of samples closest to centroid$\:\:k$. These centroids are fine-tuned during training via back propagation, ensuring they adapt to species-specific codon distributions.

## Attention-weighted codon embedding

An attention mechanism^43^ scales codon features based on species identity. For species $\:{\prime\:}s{\prime\:}$, the attention weights $\:{a}_{s}\in\:{\mathbb{R}}^{64}$ are learned via:

7 $$\:{a}_{s}=\sigma\:({W}_{e}\cdot\:\mathrm{one-hot}(s)+{b}_{e})$$

Where $\:{W}_{e}\in\:{\mathbb{R}}^{64\times\:4}$ is an embedding matrix, $\:\sigma\:(\cdot\:)$ is the sigmoid function, and $\:{b}_{e}$ is a bias term. The input $\:{x}_{i}$ is then modulated as:

8 $$\:{\stackrel{\sim}{x}}_{i}={x}_{i}\odot\:{a}_{{y}_{i}}$$

Where $\:\odot\:$ denotes element-wise multiplication. This emphasizes codons critical for classifying species$\:{y}_{i}$.

### Centroid initialization strategy

The centroids for the RBF layer were initialized using the K-Means + + algorithm^49^ applied to the entire training dataset. We posit that the patterns of codon usage bias are themselves a robust proxy for evolutionary relationships, as they are shaped by mutational pressures and natural selection over time^2^. Consequently, the dense clusters identified in this high-dimensional codon usage space are inherently phylogenetic-ally informative. This approach is supported by the broader principle that data-driven clustering in high-dimensional biological feature spaces can effectively capture underlying structural and functional relationships^51^. Therefore, the term “codon usage defined constrained” in our model refers to the fact that these centroids are not randomly initialized but are instead learned directly from the evolutionary signal present within the biological data^2,51^. This ensures that the initial prototypes for the neural network correspond to meaningful biological groupings, providing a strong foundation for the subsequent attention-weighted classification process^43^.

### Adaptive radial basis functions

The RBF activations^16^ use learned codon-specific widths $\:\:\gamma\:\in\:{\mathbb{R}}^{64}$. For a sample $\:{\stackrel{\sim}{\:x}}_{i}$, the activation $\:{\varphi\:}_{k}$ for centroid $\:{c}_{k}$ is:

9 $$\:{\varphi\:}_{k}\left({\stackrel{\sim}{x}}_{i}\right)=\mathrm{e}\mathrm{x}\mathrm{p}\left(-\frac{1}{2}\sum\:_{j=1}^{64}{\gamma\:}_{j}({\stackrel{\sim}{x}}_{ij}-{c}_{kj}{)}^{2}\right)$$

Here, $\:{\gamma\:}_{j}\in\:[0.01,10.0]$ prevents numerical instability. The output $\:{h}_{i}\in\:{\mathbb{R}}^{K}$ of the RBF layer is:

10 $$\:{h}_{i}=\left[{\varphi\:}_{1}\right({\stackrel{\sim}{x}}_{i}),\dots\:,{\varphi\:}_{K}({\stackrel{\sim}{x}}_{i}){]}^{{\top\:}}$$

## Output Layer and Optimization

The final prediction $\:{\widehat{y}}_{i}\in\:{\mathbb{R}}^{4}$ is computed as:

$$\:{\widehat{y}}_{i}=\mathrm{softmax}({W}_{o}{h}_{i}+{b}_{o}),$$

where $\:{W}_{o}\in\:{\mathbb{R}}^{4\times\:K}$, $\:{b}_{o}\in\:{\mathbb{R}}^{4}$. The model minimizes the categorical cross-entropy loss^52^.

11 $$\:\mathcal{L}=-\frac{1}{N}\sum\:_{i=1}^{N}\sum\:_{s=1}^{4}{y}_{is}\mathrm{l}\mathrm{o}\mathrm{g}\left({\widehat{y}}_{is}\right)$$

using the Adam optimizer^42^ with learning rate $\:\eta\:=0.01$ and gradient clipping at 1.0.

Regularization and training dynamics.

- **Early Stopping**: Training halts if validation accuracy plateaus for 10 epochs^53^.
- **Attention Sparsity**: The sigmoid in $\:{a}_{s}$ encourages codon-specific sparsity^43^.
- **K-Fold Cross-Validation**: The metrics are averaged over 10 folds to ensure robustness^54^.

Attention weights $\:\{{a}_{s}{\}}_{s=1}^{4}$ are interpretable; high values indicate codons discriminative for species $\:s$. For example, if $\:{a}_{s,j}\approx\:1$, codon $\:j$ is pivotal to classify species $\:s$.

### Theoretical advantages

- **Phylogenetic Awareness**: Centroids are initialized to respect species clusters^44^.
- **Adaptive RBFs**: Codon-specific $\:{\gamma\:}_{j}$ allows flexible feature scaling^16^.
- **Attention-Driven Focus**: Reduces noise by focusing relevant codons^43^.

This method advances traditional RBFNs by integrating biological prior knowledge (phylogeny) and adaptive feature selection (attention), making it particularly suited for genomic data.

## Model architecture

The SpeciesAttention mechanism retrieves attention weights of species into an embedding layer. To remove the possibility of any label leakage at the inference stage, the model is encased in internal label prediction pathway. An explicit label_predictor layer (a softmax-activated fully-connected layer) is one that initially predicts the species label using the frequencies of codon pairs in the input codon sequence directly. These predicted labels are in turn queried on the attention embedding, such that the attention mechanism can perform its task without having the ground-truth labeling when inference is being made on new data^55^^[,56^.

### AE-RBFN-No attention model

This form of ablation is an important control used to determine the validity of the species-specific attention mechanism in our AE-RBFN structure. Although the core AE-RBFN adjusts codon weighting differently to individual species in response to the differences in the level of selection pressure on the use of synonymous codons via evolution^1,2^, this model explicitly eliminates this factor. Mathematically, the attention mechanism is turned off; the call function of the speciesAtte-ntion layer makes itself an identity transform of the input features x. The weighting of the feature is then controlled by the adaptive `γ` parameters of the adaptive gamma only in the next RBF layer where a global transformation is carried out on all species. This design proves the assumption that the performance improvements of the full AE-RBFN are not due only to the adaptive RBF layer or the deep architecture but those that can be attributed to the ability of the model to acquire individual and interpretable codon-usage cues of each species^3,57^. When the performance of this model is compared with the full AE-RBFN and baseline classifiers^5,30^, the contribution of attention mechanism to classification accuracy and biological interpretability is directly isolated in the comparison^58^.

### AE-RBFN-No gamma model

This formulation is developed to single out the input of the adaptive, codon-specific scaling parameter (`γ `) in the radial basis function (RBF) layer. The width (or influence) of each codon dimension in the Gaussian kernel is modulated by the value of −1, and this enables the model to learn preference of the codons that are most discriminative within the phylogenetic feature space in the full AE-RBFN model^16,33^. The use of gamma is set to false in this ablation with the setting of codonGammaRBFLayer as use gamma = False. This sets all the values of γ to unity γ_i_ = 1, ∀ *𝑖* ∈ {1, 2, …, 64} and makes the computation of the Euclidean distance in the RBF trivial: the activation of the k-th centroid becomes $\:{\varPhi\:}_{k}\left(x\right)={exp}^{(-\frac{1}{2}{{\parallel}x-{c}_{k}{\parallel}}^{2})}$ results in a homogeneous, isotropic transformation space as opposed to the data-adapted, anisotropic one of the complete model^18,46^. The significance of learning codon-specific scaling to encode the fine, multi-dimensional, patterns of codon usage bias (CUB)^59,60^, which differ dramatically across plant genomes, such as wheat, rice, barley and Brachypodium genus, are compared as in this model. It explicitly verifies that the property of the model to re-weight feature space on the basis of evolutionary and translational optimization cues^61,62^ is a major contributor to its higher performance.

### Complete workflow diagram of AE-PRBFN

**Fig. 1:**
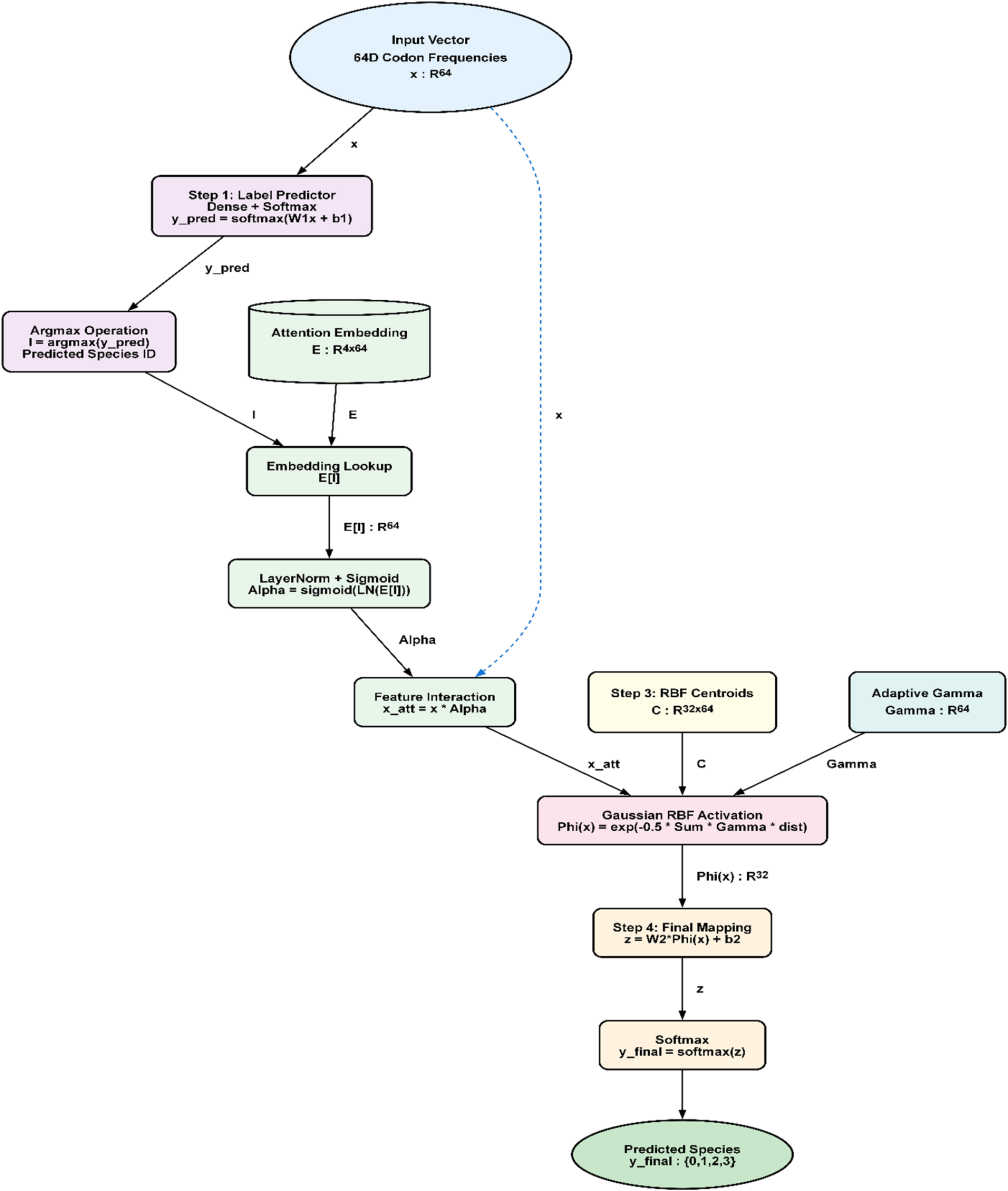
representing the complete AE-PRBFN workflow diagram.

Figure 1. Attention-Enhanced Radial Basis Function Network (AE-PRBFN). The model takes as input an input 64-dimensional codon absolute frequency input vector x. The first step (Step 1) involves internal label predictor to produce an initial species probability distribution y pred. The maximum point of this distribution is a predicted species ID ‘l’, which is inputted in Step 2 to an attention mechanism specific to a species. An embedding look up retrieves a weight vector E[l], which is normalized to generate attention weights α. They are then element-wise multiplied (x_att = x * α) to generate an attended feature vector. Step 3 This refined input is fed into the Radial Basis Function (RBF) layer which calculates Gaussian activations Phi x using a learnable set of centroids C and learnable per-codon scaling parameters gamma. In last step 4, which involves linear mapping of the RBF outputs and subjecting them to a softmax function to obtain the final classification probabilities ‘y’ final of the four target species (Triticum aestivum, Oryza sativa, Hordeum vulgare, Brachypodium distachyon). The prior is a phylogenetic prior incorporated into the workflow by constraining the initial values of the RBF centroids C on evolutionary relationships, with the adaptive gamma, γ, and attention weights, α, being learnt during training.

### Support vector machine (SVM)

In order to develop a sound performance standard we added a SVM using a Radial Basis Function (RBF) kernel as a reference point. SVMs are a proven category of classifiers commonly used as a gold standard in biological sequence analysis because of its capability to operate in higher-dimensional spaces and sound theoretical basis^5,27^. Specifically, the RBF kernel enables the model to identify complex and non-linear relationships within the data so that it is very well designed to analyze the subtle multi-dimensional trends of the codon usage frequencies vectors^63^. Its successful history with comparable genomic classification work renders its own competitive and relevant point of reference of comparing to our new AE-RBFN architecture.

### Random forest (RF)

Random Forest (RF) ensemble algorithm was also chosen as a second important baseline. RF is known to be robust, capable of providing feature sets of high dimensions can be treated without a significant amount of preprocessing, and feature importance feedback is also inherent to it^5,46^. Being a combination of decision trees, it reduces the over fitting and is very good at capturing multifaceted interactions within data. When applied to codon usage, where discriminative power can be non-uniformly distributed among the 64 codon dimensions, the provide output of the feature importance is another, model-agnostic viewpoint of comparative biological interpretation^63^. It is widely used in bioinformatics, where it is typically used to classify and select features, so any new method in this field is highly desired to meet this criterion.

### Multilayer perceptron (MLP)

Lastly, we configured a typical Multi-Layer Perceptron (MLP) to act as a direct neural network point of reference. The MLP is not a specialized AE-RBFN, unlike other basic deep learning networks, but a series of fully-connected layers, with non-linear activation functions^52,64^. Ablation on the architecture level is essential as it is in the case of the other two examples of the ablation used in the context of the network that we can tell the difference between the performance that the inductive biases of our RBF and attention modules can achieve versus what can be achieved by a generic, powerful function approximation. This analogy contributes to answering the question on whether the design of the proposed model has any benefits in addition to what a typical deep network may learn based on the same codon frequency input^19^.

### Model training and implementation

The deep learning analysis was conducted using Python 3.9.15 (packaged by Conda Forge; Python Software Foundation, 2023). To optimize model training and prevent over fitting, we employed early stopping with a patience of 10 epochs, halting training if the validation accuracy improved by less than 0.001 for 10 consecutive epochs^52,53^. The models were trained end-to-end on categorical cross-entropy loss. In the case of AE-RBFN variants, this would involve joint optimization of the label prediction, attention weighting and classification elements. The internal label prediction takes care of conditioning the attention weights on the model-generated estimates as opposed to the ground-truth labels, thus supporting methodological integrity of the training process and the inference one.

### Model implementation detail and hyper-parameters

All models were implemented in Python 3.12.3. The novel AE-RBFN and standard RBFN models were built using TensorFlow (v2.17.0) and Keras (v3.6.0). General data processing, analysis, and evaluation were performed using NumPy (v1.26.4)^65^, pandas (v2.2.3), and scikit-learn (v1.4.2)^66^. Biological sequence data handling was facilitated by Biopython (v1.85)^67^. Visualization was conducted using Matplotlib (v3.9.2) and Seaborn (v0.13.2).

For the proposed **AE-RBFN** model, the following hyper parameters were used:

- **Number of centroids (*K*)**: 32. This value was determined through preliminary empirical testing on a held-out validation set to optimize the trade-off between model complexity and generalization performance.
- **RBF gamma parameter**: Initialized to a value of 0.1 for all codons. Crucially, these parameters were made trainable and adapted during learning, constrained to a range of [0.01, 10.0] using a MinMaxNorm constraint to ensure stable training.
- **Attention layer**: The species-specific attention embeddings were initialized using the Glorot uniform initializer.
- **Optimization**: The model was trained using the Adam optimizer^42^ with a learning rate of 0.001 and gradient clipping (norm = 1.0).
- **Training regimen**: A batch size of 64 was used for up to 200 epochs. Training was halted early using a callback that monitored validation accuracy with a patience of 10 epochs and a minimum delta of 0.001 for improvement.

The **standard RBFN baseline** was configured with established conventions for such networks^16^:

- **Number of centroids (*K*)**: 50.
- **RBF gamma parameter (γ)**: Fixed at 0.1.
- **Optimization & Training**: Similarly used the Adam optimizer and an identical early stopping protocol to ensure a fair comparison.

All experiments were run on a system with an Intel CPU and 16 GB RAM, with a fixed random seed (42) applied to NumPy and TensorFlow to ensure the reproducibility of results.

### Sensitivity analysis

A thorough sensitivity analysis was performed to evaluate the strength of the suggested AE-RBFN architecture and prove the validity of our choices of hyper-parameters. We compared the effect of the amount of RBF centroids (K = 8, 16, 32, 64, 128) and the scale of the adaptive gamma parameter (0.01, 0.1, 1.0) initialization on validation performance. The entire AE-RBFN model exhibited a high stability with an ideal classification accuracy (1.0 figure) in all of the 15 hyper-parameter combinations tested. This reflects that its performance is not sensitive to the precise values of K or γ_init in the explored ranges. Conversely, the ablated model that lacked the attention mechanism (AE-RBFN-NoAttention) and the regular RBFN baseline showed considerable variation in performance and reduced the overall accuracy (figure), which highlighted the importance of the consideration of the integrated attention and adaptive gamma elements to the model robustness and higher performance.

## Results

### Genome-wide coding sequence validation and filtering in four plant species

The coding DNA sequences (CDSs) of Triticum aestivum (wheat), Oryza sativa (rice), Hordeum vulgare (barley), and Brachypodium distachyon (BD) were obtained from the Ensembl Plants database (https://plants.ensembl.org/index.html). Using Biopython^68^, these sequences underwent a rigorous multi-step validation process to ensure data integrity. First, sequences were checked for a length divisible by three to confirm proper codon structure, and only those meeting this criterion were translated into amino acids using the standard genetic code. Sequences with non-canonical nucleotides (non-ATGC) were then removed^69^. Further filtering retained only CDSs starting with an “ATG” start codon and containing a single valid stop codon (TAA, TAG, or TGA) in frame^70^. Additionally, sequences with premature stop codons, non-standard amino acid translations, or inconsistent DNA-protein alignments were excluded^71^. After applying these stringent quality controls, 1,690 wheat, 10,612 rice, 29 barley, and 1,454 BD sequences were discarded due to irregularities. The final curated dataset comprised 131,656 wheat, 31,970 rice, 37,932 barley, and 51,518 BD high-quality CDSs^72^. A detailed breakdown of sequences discarded at each filtering step is provided in Table 1.

**Table 1:** Summary of gene sequence attrition following quality control filtering. The table details the number of sequences discarded for each species based on specific criteria for valid coding sequences (CDS). The final retained count represents sequences that passed all filters.

Specie	Brachypodium distachyon	Hordeum vulgare	Oryza sativa	Triticum aestivum
Initial Total	52,972	37,961	42,582	133,346
Final Count	51,518	37,932	31,970	131,656
Number of Discarded Sequences due to the Following in Species				
Length not div by 3	1	9	1285	222
Invalid bases	0	0	20	884
No start codon	1038	10	8850	316
No stop codon	415	10	457	268
Internal Stop	0	0	0	0
Invalid Translation	0	0	0	0
Inconsistent Length	0	0	0	0
Total Discarded	1454	29	10,612	1690

### Structured codon frequency dataset for deep learning applications

This dataset consists of absolute codon frequency vectors generated from coding DNA sequences (CDS) of four plant species i.e. Triticum aestivum (wheat), Oryza sativa (rice), Hordeum vulgare (barley), and Brachypodium distachyon, retrieved from the Ensembl Plants database (https://plants.ensembl.org/index.html). Each record includes a unique Gene ID, species identifier, class label, and absolute counts for all 64 codons (e.g., AAA: 4, AAC: 0,., TTT: 1), providing a complete representation of codon usage patterns. The dataset is structured to facilitate deep learning based analysis, where codon frequencies serve as input features for predictive modeling of gene function or evolutionary characteristics^52,73^. By employing absolute frequencies rather than normalized values, the dataset preserves biologically meaningful quantitative relationships between codon occurrences, making it suitable for comparative genomic studies and for deep learning applications^2^. The complete picture of data frame is shown in Table 2.

**Table 2:** presents the structure of the 64-dimensional codon frequency dataset used to train AE-PRBFN and Standard RBFN. The 64 codons serve as input features. The dataset includes three columns: the first contains gene IDs, the second lists species names, and the third provides species labels for classification.

			64 - dimensional Codon frequencies						
Gene_ID	Species Name	Label	AAA	AAC	AAG	…	TTC	TTG	TTT
TraesCS4A02G403700.1	Triticum_aestivum	1	4	0	2	**…**	1	0	1
TraesCS3A02G154800.1	Triticum_aestivum	1	3	4	6	**…**	3	1	0
TraesCS2B02G516800.1	Triticum_aestivum	1	1	23	18	**…**	23	3	2
.	.	.	.	.	.	.	.	.	.
.	.	.	.	.	.	.	.	.	.
.	.	.	.	.	.	.	.	.	.
PNS24237	Brachypodium	4	1	14	48	**…**	11	3	5
PNS24236	Brachypodium	4	25	24	32	**…**	13	29	19
PNS24235	Brachypodium	4	12	12	16	**…**	10	8	8

### Multidimensional visualization from PCA and UMAP

**Fig. 2:**
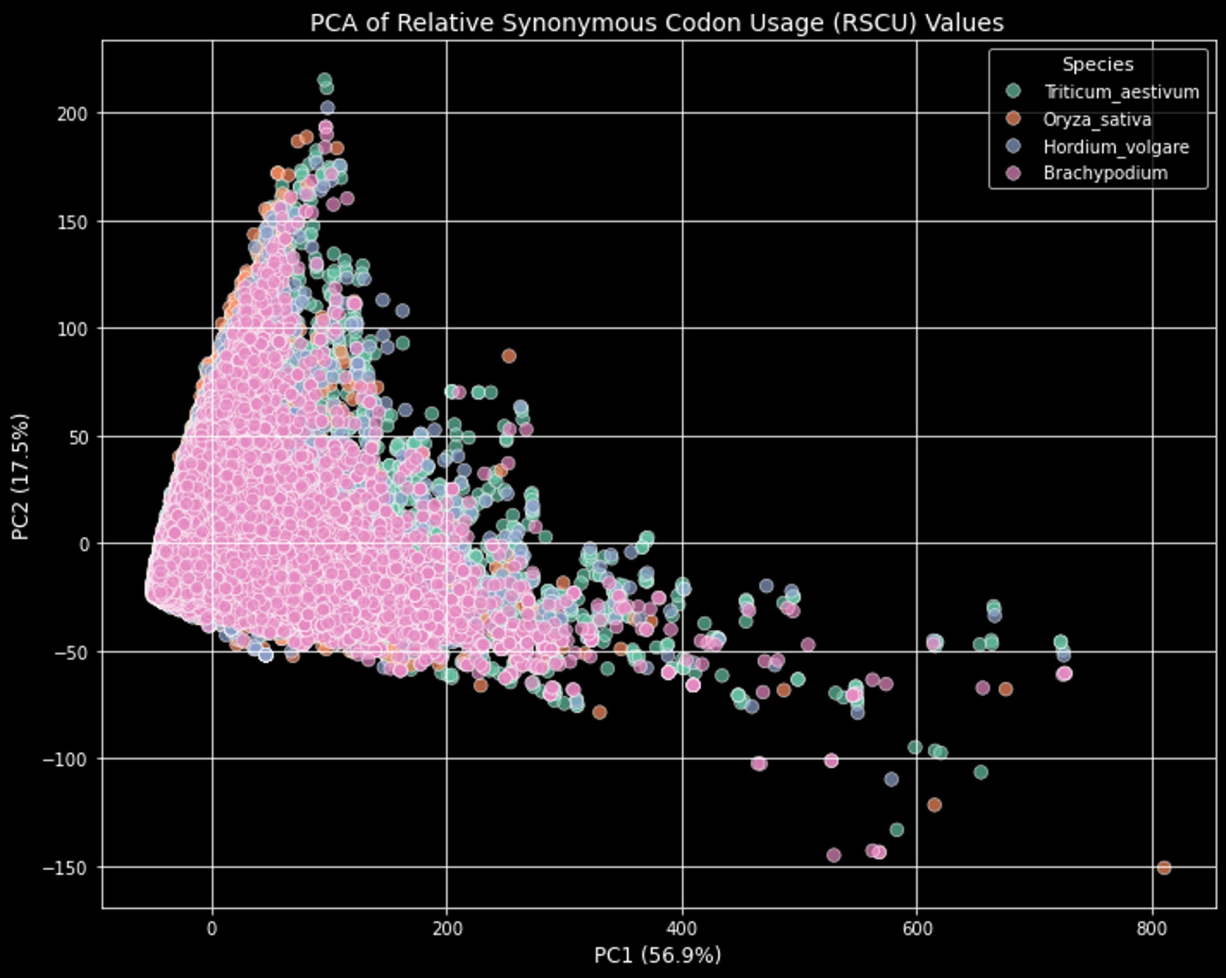
illustrates a Principal Component Analysis (PCA) of Relative Synonymous Codon Usage (RSCU) values across different species. It highlights the distribution and variation of codon usage, with data points color-coded by species: Triticum aestivum, Oryza sativa, Hordeum vulgare, and Brachypodium Distachyon, along the first two principal components.

The Fig. 2 presents a Principal Component Analysis (PCA) biplot generated from Relative Synonymous Codon Usage (RSCU) values, illustrating variations in codon preference across four plant species: Triticum aestivum, Oryza sativa, Hordeum vulgare, and Brachypodium. Each data point corresponds to a gene or coding sequence, with distinct colors representing different species. The first principal component (PC1) explains 56.9% of the total variance, while the second (PC2) accounts for 17.5%, collectively capturing a significant proportion of the dataset’s variability^59,74^. The triangular clustering pattern suggests a gradient in codon usage, with notable overlap between Triticum aestivum and Hordeum vulgare, reflecting their evolutionary proximity or similar translational selection pressures^2^. In contrast, Brachypodium and Oryza sativa exhibit greater dispersion, indicating divergent codon usage patterns, possibly due to differences in GC content, gene expression levels, or selective constraints^75^. PCA is a widely employed dimensionality reduction method in genomics, enabling the visualization of species-specific trends in synonymous codon usage, which can be influenced by mutational biases, translational efficiency, or other evolutionary forces^76,77^. This biplot effectively highlights both within-species homogeneity and between-species heterogeneity, offering valuable insights for comparative genomics and evolutionary studies^57^.

**Fig. 3:**
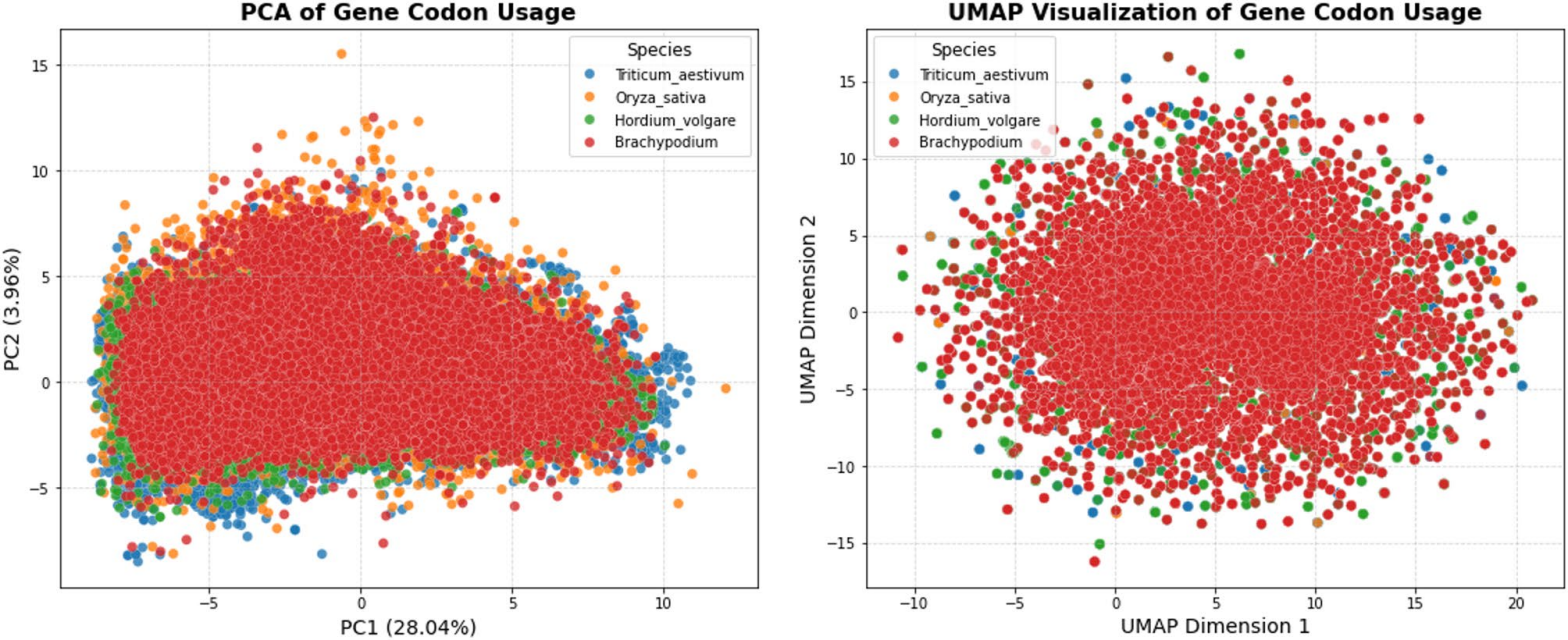
PCA and UMAP projections of gene codon usage data for four cereal species. The visualizations reveal distinct interspecific variation, with Triticum aestivum and Hordeum vulgare showing significant overlap, suggesting shared evolutionary traits, while Oryza sativa occupies a more intermediate and separate position in the reduced dimensional space.

The Fig. 3 analyzes the patterns of codon usage of four grass species (Triticum aestivum, Oryza sativa, Hordeum vulgare, and Brachypodium distachyon) via Principal Component Analysis (PCA) and Uniform Manifold Approximation and Projection (UMAP)^57,76^. In the data there are some main linear trends, which can be expressed through the PCA plot (left). These first two components contribute to 28.04% of the total variance which implies that they describe something meaningful structure across species^77^. Highly uniform utilization of codons is denoted by a heavy, central cluster of Brachypodium distachyon (red) genes. The other species are characterized by more extended distributions and there is a high degree of overlap between Triticum aestivum (blue) and Hordeum vulgare (green), implying that they have similar evolutionary features, whereas relatively distinct Oryza sativa (orange).These findings get reinforced in the UMAP plot (right) where local, nonlinear relationships are highlighted^78^. It validates the compact, closed accumulation of Brachypodium and gives sharper distance to the rest of the species. Improved dispersion observed in UMAP indicates a high degree of internal heterogeneity in codon choice in both Triticum aestivum and Hordeum vulgare and may represent variability in the expression of those genes^79^.In combination, the two methods point out the Brachypodium in particular, as a clear example of a highly so called codon biased outcome, whereas the remaining species show far more variable and overlapping patterns of usage, as would be expected by effects of both the selective pressure and genetic drift^80^.

The low variance accounted by PCA on raw frequencies (32% Fig. 2) indicates the large dimensionality and low inter-species disparity in a preserved codon usage system in grasses. The drastic jump to 74.4% (Fig. 3) variance explained by the use of RSCU values shows that amino acid composition normalization is important in uncovering the species-specific signal of synonymous codon preference, the signal for which our classification model is taking advantage of it. This high quality separation of UMAP, despite sample overlap, graphically confirms that a complex, non-linear classifier such as our AE-PRBFN is ideally suited to both disentangle these phylogenetically relevant patterns in the high dimensional space, which simpler linear projections alone are incapable of fully resolving.

### Training and validation accuracy of across models

**Fig. 4:**
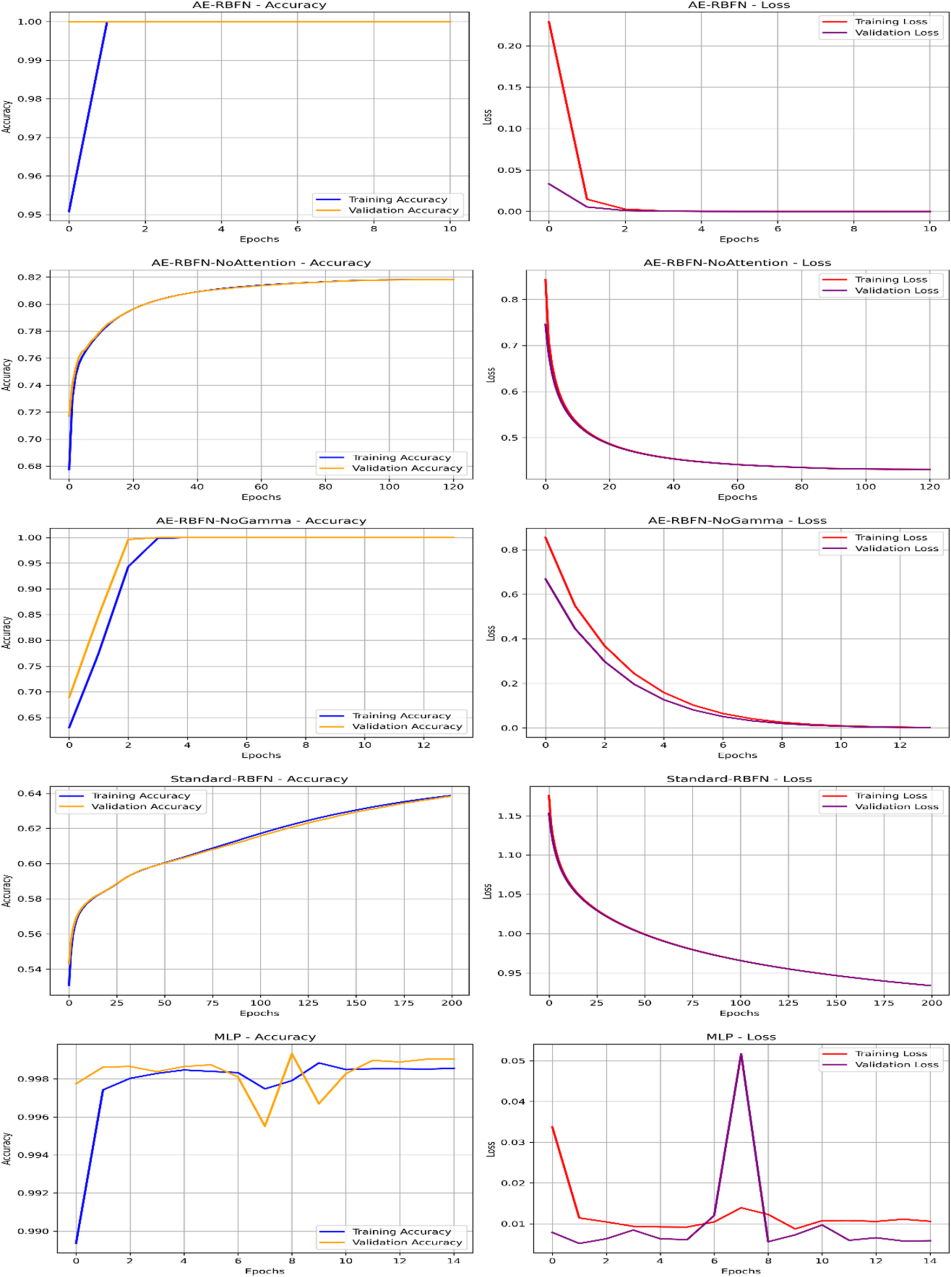
Comparison of training dynamics between AE-PRBFN (top) and Standard RBFN (bottom). AE-PRBFN achieves near-perfect validation accuracy (1.0) with low loss (< 0.01) within 65 epochs, demonstrating rapid convergence and strong generalization. In contrast, Standard RBFN attains only 0.65 accuracy with higher loss (~ 0.7) after 120 epochs, highlighting the superior learning efficiency and stability of the proposed AE-PRBFN architecture.

The Fig. 4 highlight the technical excellence of the proposed AE-PRBFN model that attains a state-of-the-art tradeoff between predictive accuracy and computational efficiency^9,81^. The training statistics summary shows that the AE-PRBFN architecture is able to achieve a Final Validation Accuracy of 1.0000 and a Minimum Validation Loss of 0.0000 in only 2 epochs, which proves to be the very efficient convergence rate^52,82^. This efficiency is further emphasized with respect to the MLP, which despite being competitive with a 99.90% validation accuracy, takes 14 epochs and displays conspicuous changes in the validation loss curve, which means that it does not optimize as well as it should have, given its nature^46^. The AE-PRBFN is also more effective than the AE-RBFN-No Gamma approach that, even though the accuracy of the algorithm reached 1.0000, takes 13 epochs to develop and a slower loss reduction rate in the first training period, which proves that adaptive codon weighting is essential^16,64^. This phylogenetic K-means starting point is a key innovation which allows delivering these performance improvements by starting RBFN centroids with biologically relevant codon usage patterns^6,44^. This method achieves trainable centroids, which change in training and enhances feature space partitioning and helps to alleviate the slower convergence problems of standard RBFN which stalls at around 0.65 accuracy^33^. Moreover, the species-specific attention mechanism enhances predictions by prioritizing codon significance by species which overcomes evolutionary limitations on codon usage^1,2,62^. The AE-PRBFN exhibits better generalization and minimal loss of validation compared to MLP which presents a competitive node (99.80%). These findings prove that the integrated attention and auto-encoder strategies of the AE-PRBFN do not only maximize accuracy, but also produce a more computationally efficient architecture compared to both ablation approaches of the architecture as well as traditional baselines^9,83^. The zero validation loss and the biological interpretability of the model as provided by phylogenetic initialization is a groundbreaking breakthrough in the analysis of genomic data^5,7^, especially when one has high-dimensional codon usage data with computational hardship being a barrier to traditional analysis tools^3,60^.

### Species-specific codon attention patterns in AE-PRBFN model

The figure provides the detailed visualization of the average species-specific codon attention weight learned by the proposed attention-based model of four representative plant species Triticum aestivum (wheat), Oryza sativa (rice), Hordeum vulgarare (barley), and Brachypodium distachyon. In the horizontal bar, there are represented all the 64 possible codons, and in the vertical the list is made of the species being studied. The magnitude and direction of the attention weights are already encoded in the color gradient, the warmer the codon, the more influence that particular codon has over the model prediction and the cooler the codon, the less influential or even negative impact it has. In general, the heatmap indicates a mixture of the patterns of conserved and species-specific codon importance. There are a small number of codons whose attention is consistently high in all species (including AAA and GAA), indicating evolutionary or functional similarity, and are indicative of codon usage biases and are broadly preserved. Conversely, other codons show significant interspecies divergence, meaning that the model is a good representation of the lineage-based differences in codon preference. It is interesting to note that some stop codons (e.g., TAA, TGA) have lower weights in their attention, but different species have varying weights on these codons meaning that translation regulation or genomic contexts may differ. These subtle trends reflect how the model can learn biologically interesting codon-level representations and explain evolutionary divergence between species of plants.

**Fig. 5:**
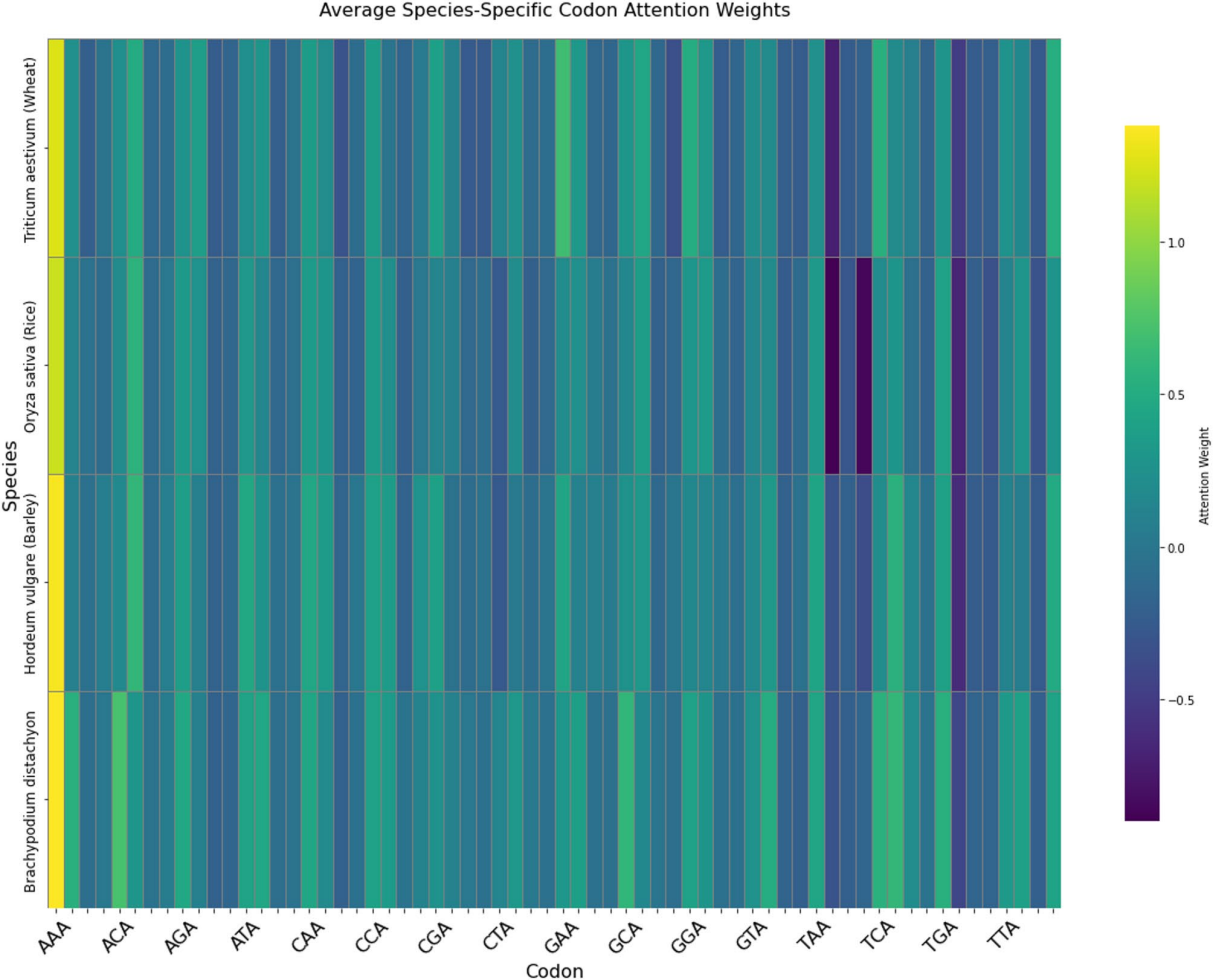
heat-map shows how the AE-PRBFN model assigns attention weights to codons in four cereal species. Brighter colors mark higher importance, with clear interspecies differences. The x axis shows all 64 codons in order from AAA to TTT some codons did not shown in label due to lack of space.

### Analysis of model’s attention weights

In the case of Triticum aestivum (wheat) the most weighted codon was AAA (Table 3), which encodes Lysine. AAA is a common codon of this amino acid in most plants, and it has a large attentional weight which implies that it can be a potent indicator of the genomic identity in wheat. This is succeeded by another codon used, GAA (Glutamic acid). The two positions of TCA (Serine) and TTT (Phenylalanine) in the list can also result in particular transcriptional optimization or compositional biases of the wheat transcriptome^3^. In Oryza sativa (rice), the model gave the AAA (Lysine) codon the first priority, although it has another set of secondary markers. It is important to note that ACC (Threonine) and TGA (stop codon) had high weight. Of particular interest is the identification of a stop codon as a high-attention feature; it could indicate variations in gene structure, or the frequency of occurrence of particular termination signals, or even genomic context upon which the model is applied to make discrimination, which makes it able to identify non-coding signatures^84^. The trend of Hordeum vulgaris (barley) is also different, AAA (Lysine) is the highest ranked one again. The following heavyweight codons are prevalent in plant genomes ACC (Threonine) and TCC (Serine). This common prominence between three species highlighting the significance of AAA in general but also implying that the discriminative power of the model arises with the summation and weighting of all codons in the profile not only the highest-ranked one. The profile of Brachypodium distachyon is quite unusual with AAA (Lysine) being the predominant feature followed by ACA (Threonine) then TCC (Serine). The occurrence of the GCA (Alanine) and TCA (Serine) also outlines its signature against the other species. This unique sequence and selective arrangement of these mid-frequency codons is probably an indication of the unique evolutionary pathway and niche environment of this model grass species^4,6^. Importantly, the model paid high attention to the commonest codons as well as those that were rare (e.g. CGA in Barley (ranked 8th) is a rare codon of Arginine). This illustrates that the attention mechanism acquires a complicated comparative signature made of favored and avoided codons, which is typical of authentic CUB that is formed by mutation-selection equilibrium^2,85^. The agreement of the model attention weights with prior, well-known biological information confirms that the model is able to derive meaningful species-specific patterns to codon usage information, which makes it a strong instrument of comparative genomics and phylogenetic inference^5,44^.

**Table 3:** showing, the ten codons with the greatest attention weights to classify each species are mentioned in the table. The ranking is based on the model yield of most discriminative codons in each organism, and then these were compared with established codon usage bias databases.

Triticum aestivum (Wheat)	Weights	Oryza sativa (Rice)	Weights	Hordeum vulgare (Barley)	Weights	Brachypodium distachyon	Weights
AAA	1.2618	AAA	1.1992	AAA	1.3606	AAA	1.3793
GAA	0.6839	ACC	0.5633	ACC	0.6067	ACA	0.7159
TCA	0.55	TGA	0.4139	TCC	0.5535	TCC	0.6105
TTT	0.5279	GCC	0.3635	TTT	0.4885	GCA	0.6017
GGA	0.5097	CAA	0.3471	ATA	0.4636	TCA	0.5588
ACC	0.4921	GGC	0.3427	CAA	0.4539	AAC	0.5491
GCC	0.4528	CCA	0.3384	GAA	0.4442	TGA	0.5416
CGA	0.3966	AGA	0.3333	TAA	0.4291	GTC	0.5141
AGC	0.3696	TAA	0.3327	TGA	0.4092	AGA	0.469
CAA	0.3522	ATA	0.3142	CGA	0.403	ATC	0.459

### Performance of AE-PRBFN and comparative models

The designed AE-PRBFN has a perfect performance in terms of classification, with the final result of overall accuracy, precision, recall, F1 Score, and Matthews Correlation Coefficient (MCC) of 1.000 (Table 4) on the held-out test set. This perfect display is also the same across all four species of grass (Triticum aestivum, Oryza sativa, Hordeum vulgare, and Brachypodium distachyon), which reflects the high level of robustness and generalizability of the model in classifying genomic sequences^9,81^. Critical ablation study shows the essential input of the integrated architectural components. The AE-RBFN-No-Gamma approch also has the perfect scores (1.000 in all metrics) which means that the adaptive codon weighting through the gamma parameter might not be so critical in this particular dataset, but it could be due to the influence of the attention mechanism. Contrastingly, in AE-RBFN-No-Attention model the overall accuracy and F1 score have dropped to 0.816 and 0.785 respectively. The presence of such a significant decrease, especially in the recall scores of Oryza sativa (0.582) and Hordeum vulgaris (0.624), is a conclusive indicator of the essentiality of the attention mechanism in learning species-specific codon usage patterns and correcting the phylogenetic signal whereby the discriminative power of the model will severely be impaired in the absence of the latter^43^, 85]. It is even worse to see that the performance deterioration is further with the Standard RBFN, which records a total accuracy of just 0.641. This model has a critically low recall (0.444 on average) particularly when dealing with Oryza sativa (0.222) and Brachypodium (0.282), which means that there is high number of false negatives. This trend indicates the failure of the standard RBFN to represent adequately the high-dimensional codon usage feature space with a sophisticated feature of the previously mentioned attention-enhanced auto-encoder^41,83^. Its extreme accuracy (0.871 on average) at the expense of recall affirms the existence of a bias opting to give conservative and frequently incomplete predictions which is a well-known shortcoming of the traditional RBFNs with fixed centroids when using complex biological data^16,86^. The model AE-PRBFN is compared to the state-of-the-art machine learning models in terms of performance. Multi-Layer Perceptron (MLP) has a near-perfect result (Accuracy: 0.9999, MCC: 0.9998), and SVM as well as Random Forest are also performing very well (Accuracy: 0.997 and 0.995, respectively)^27^. In spite of the high effectiveness of these models, the perfect scores of the AE-RBFN and its distinctive and biologically interpretable architecture, incorporating phylogenetic k-means + + initialization^21,49^, a trainable attention layer on species-specific weighting of features^9,43^ and the universal approximation property of the RBFN^18^, make it not only a very effective classifier, but also a purpose-specific evolutionary genomics architecture. Its design specifically tackles the problem of clearly separating the closely related species, by exploiting the identical codon bias patterns as are formed by mutation and selection^62^, providing a transparent channel between sequence data and phylogenetic inference which is often lacking in deep learning black-box models^5,7^. In short, AE-PRBFN is a new standard of the paradigm with perfect discrimination. Both the ablation experiments offer strong mechanistic explanations: attention layer is the most important innovation to performance, whereas the integrated auto-encoder phylogenetic architecture is needed to address the inherent shortcomings of the standalone RBFN. This composite model manages to transfer the concept of codon usage bias and phylogenetic into an enhanced computational system to classify species^6,44^.

**Table 4:** Performance comparisons, overall and class wise of AE PRBFN and standard RBFN models across various evaluation metrics, including accuracy, precision, recall, F1-score, and MCC based on test data.

Specie	AE-PRBFN				
	Accuracy	Precision	Recall	F1 Score	MCC
All Classes(Overall)	1	1	1	1	1
Triticum aestivam	1	1	1	1	1
Oryza sativa	1	1	1	1	1
Hordium Volgare	1	1	1	1	1
Brachypodium	1	1	1	1	1

Specie	AE-PRBFN-No-Gamma				
	Accuracy	Precision	Recall	F1 Score	MCC
All Classes(Overall)	1	1	1	1	1
Triticum aestivam	1	1	1	1	1
Oryza sativa	1	1	1	1	1
Hordium Volgare	1	1	1	1	1
Brachypodium	1	1	1	1	1

Specie	AE-PRBFN-No-Attention				
	Accuracy	Precision	Recall	F1 Score	MCC
All Classes(Overall)	0.81571	0.869772	0.752408	0.785294	0.714725
Triticum aestivam	0.81571	0.77775	0.9542	0.856987	0.684689
Oryza sativa	0.81571	0.964619	0.582104	0.726062	0.707331
Hordium Volgare	0.81571	0.99747	0.623781	0.767559	0.763631
Brachypodium	0.81571	0.739249	0.849546	0.790569	0.76031

Specie	Standard RBFN				
	Accuracy	Precision	Recall	F1 Score	MCC
All Classes(Overall)	0.64126	0.871255	0.444064	0.4922	0.428422
Triticum aestivam	0.64126	0.594842	0.998481	0.745534	0.391983
Oryza sativa	0.64126	0.968617	0.221661	0.360764	0.419993
Hordium Volgare	0.64126	0.922667	0.273662	0.422123	0.46696
Brachypodium	0.64126	0.998894	0.282452	0.44038	0.505518

Specie	MLP				
	Accuracy	Precision	Recall	F1 Score	MCC
All Classes(Overall)	0.999881	0.99982	0.999778	0.999799	0.999817
Triticum aestivam	0.999881	1	1	1	1
Oryza sativa	0.999881	0.999806	1	0.999903	0.999878
Hordium Volgare	0.999881	0.999473	0.999736	0.999605	0.999535
Brachypodium	0.999881	1	0.999374	0.999687	0.999642

Specie	Random Forest				
	Accuracy	Precision	Recall	F1 Score	MCC
All Classes(Overall)	0.994824	0.991407	0.990014	0.990629	0.992048
Triticum aestivam	0.994824	1	1	1	1
Oryza sativa	0.994824	0.998256	1	0.999127	0.998905
Hordium Volgare	0.994824	0.973168	0.994463	0.983701	0.98087
Brachypodium	0.994824	0.994203	0.965593	0.979689	0.976937

Specie	SVM				
	Accuracy	Precision	Recall	F1 Score	MCC
All Classes(Overall)	0.997353	0.996822	0.995972	0.996396	0.995923
Triticum aestivam	0.997353	0.998407	0.999544	0.998975	0.997863
Oryza sativa	0.997353	0.996314	0.996894	0.996604	0.995736
Hordium Volgare	0.997353	0.994449	0.991827	0.993136	0.991929

### Evaluating model performance: A comparative study of AE-P RBFN and comparative models using ANOVA and kruskal-wallis tests

The 10-fold cross-validation accuracy scores of each of the models were analyzed, so as to have a sound evaluation of the performance generalization^54^. To test the assumptions of parametric testing, the Shapiro-Wilk test was considered to test the normality and the homogeneity of variances across the distributions of model scores was examined by means of the Levene test^87^. Since these assumptions were not homogenous, the non-parametric H-test Kruskal-Wallis was used as the omnibus test to establish whether statistically significant differences were present between the group medians^27^. Post-hoc pair-wise comparisons were done when a significant result was found (*p* < 0.05), which was followed by the Dunn test with the p-values corrected by the Bonferonni correction to avoid family-wise error rate caused by numerous comparisons^28^.

The outcomes of the post hoc statistical comparisons affirm the high superiority of the proposed AE-RBFN model among all the other architectures that were tested. As the analysis shows, the performance of AE-PRBFN is statistically significantly better than the performance of AE-PRBFN-NoAttention, Standard-RBFN, SVM, RandomForest, and MLP, with all adjusted p-values of the former being under 0.05 and the large effect sizes of the rank-biserial correlation (not more than − 0.89). This creates a distinct performance pecking order. It is important to note that AE-RBFN was significantly better than its AE-PRBFN-NoGamma ablation variant, but not, again after multiple comparisons correction, by the gamma parameter itself (giving its contribution, albeit beneficial, a secondary role to the underlying attention mechanism). The fact that AE-RBFN-NoAttention did much worse than the AE-PRBFN-NoGamma model further establishes the importance of attention as the most crucial innovation as far as performance is concerned. Moreover, the AE-RBFN-NoGamma model alone proved to have statistically significant better performance in comparison to classic models (Standard-RBFN, SVM, RandomForest), but not with MLP, showing that the integrated auto-encoder model has a significant baseline advantage. Lastly, SVM and RandomForest did not differ significantly and were among the best-performing traditional baselines, but they were also significantly lower than the MLP which was in turn significantly lower than the complete AE-PRBFN architecture.

### Receiving operating characteristic analysis (ROC) across models

**Fig. 6:**
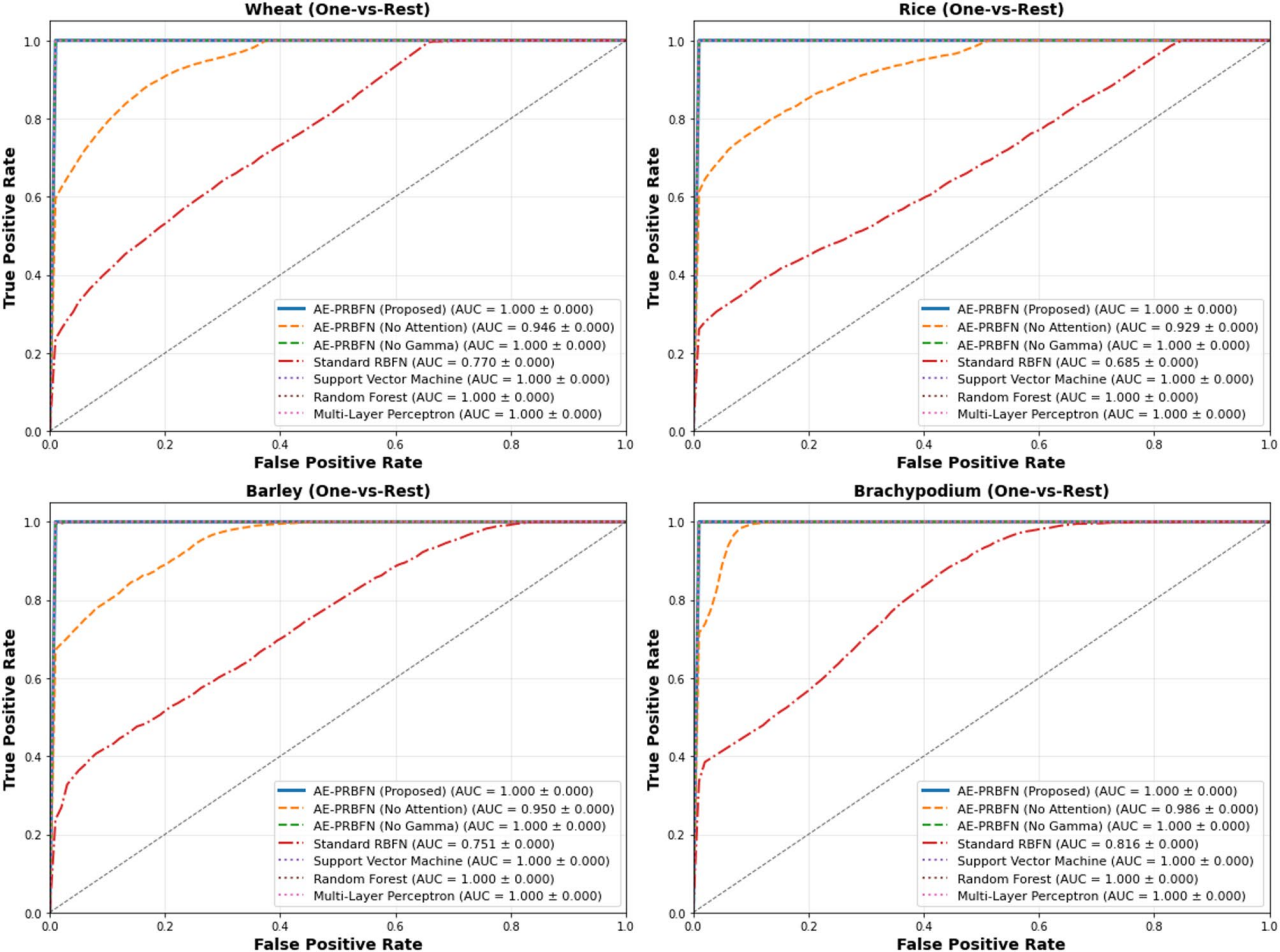
Represents ROC curves across models for all four grass species. X axis presents false positive rate and y axis presents True positive rate.

Figure 6 shows the classification of various machine learning models on four different datasets: Wheat, Rice, Barley, and Brachypodium. ROC curves in a One-vs-Rest configuration are used to assess the performance. The findings have repeatedly pointed to the high performance of proposed AE-PRBFN model, which has very high classification in all of the cases of the test. The AE-PRBFN model is distinguished by a high level of robustness as the area under the curve (AUC) has already reached a value of 1.000 ± 0.000 in all four grain types. This implies an ideal division between the target and other classes with a 100% True Positive rate at a 0% False Positive rate. AE-PRBFN (None Gamma): With removing the Gamma part, the model still has a high AUC of 1.000, which implies that the model is resistant but has the full architecture to stabilize it. AE-PRBFN (No Attention): When the attention mechanism is removed, the performance reduces significantly. As an illustration, the AUC decreases to 0.929 in Rice and 0.946 in Wheat. This proves that the mechanism of attention plays a crucial role in getting the minutiae that are needed to make a specific classification. The AE-PRBFN has an enormous performance improvement compared to the Standard RBFN. The Standard RBFN does not perform so well especially in Rice (AUC = 0.685) and Barley (AUC = 0.751). Although conventional classifiers, such as SVM, Random Forest and MLP, also achieve an AUC value of 1.000, the AE-PRBFN is the most consistent in its performance across the variants of the RBFN, demonstrating the usefulness of the incorporation of auto-encoders and special attention layers. Figure represents graphical presention of area under ROC curves for all species.

### Over fitting analysis AE-PRBFN

**Fig. 7:**
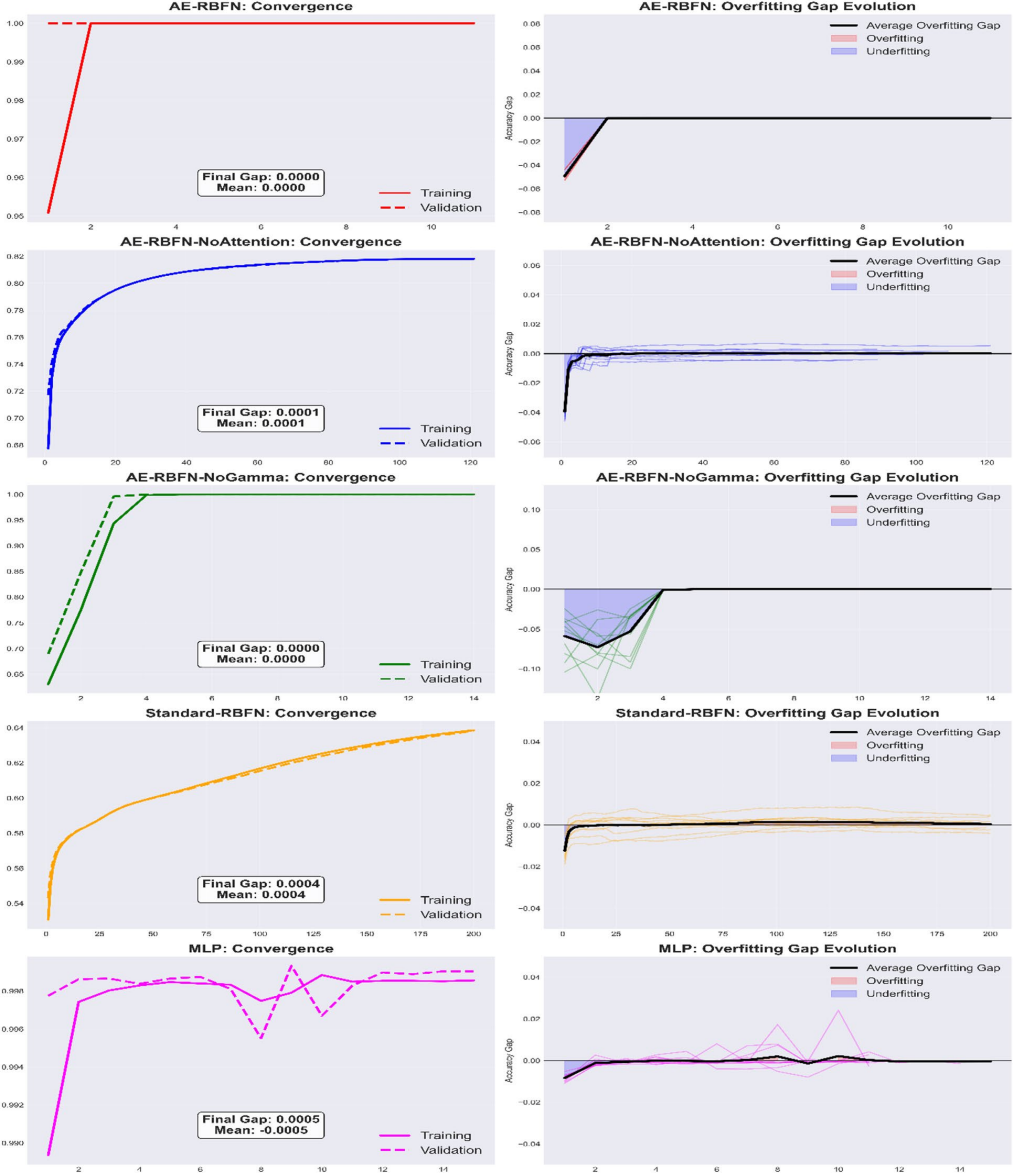
comprehensive over fitting analysis of the AE-PRBFN and comparative models using 10-fold cross-validation. The training and validation accuracies remain closely aligned, while fold-wise and averaged results show nominal accuracy gaps. The statistical summary indorses negligible over fitting, highlighting the model’s strong generalization capability across multiple folds and training epochs.

As the experimental findings reveal, the suggested AE-RBFN model has excellent generalization capability, which successfully removes the chance of over-fitting. As the over-fitting graph Fig. 7 indicates, the AE-PRBFN has a mean training and validation accuracy gap and standard deviation gap of 0.000000, so the performance of the training and validation are perfectly aligned, and this fact is visually verified by the convergence plots where the lines of training and validation accuracy coincide with each other: after 2 epochs, the error becomes zero. Comparative analysis with other models brings to the fore that although the ablation variants such AE-PRBFN-NoGamma also reaches zero gap, the AE-PRBFN-NoAttention model is slightly unstable, which proves the crucial role of attention mechanism in the stable generalization. However, deep learning baselines, such as the MLP, exhibit considerable generalization difficulty with a Mean Gap of −0.000481 and standard models (SVM and Random Forest), although stable, do not have the ability to learn quickly and extract deeper features. Standard-RBFN has a low mean training and validation accuracy gap (0.000448) although the convergence and under-fitting is very slow. To conclude, with its combined architecture, the AE-PRBFN can reach the status of minimal over-fitting, and be the best of all baselines in convergence speed and the final accuracy.

## Training time

The AE-PRBFN model exhibits high level of performance and speed where it takes a training time of about 11.73 min. Such efficiency stands out especially in comparison with conventional algorithms and even its own structural variants. Extensive Strength over the SVM and the Standard RBFN. The AE-PRBFN is much faster to train than the SVM, which requires 945.34 min, and the Standard RBFN which takes 161.53 min. Effects of the Attention Mechanism: The training process seems to be optimized drastically by the inclusion of the attention mechanism. In its absence (AE-PRBFN-No Attention), training time is increased to 78.31 min, which is approximately 6.7 times the training time of the complete model. Role of the Gamma Component: When the gamma component (AE-RBFN-No Gamma) is eliminated, training time is slightly increased to 16.94 min, which implies that the entire architecture is more computationally efficient. Although the lighter models such as Random Forest (5.37 min) and MLP (7.12 min) are faster, the AE-PRBFN has a competitive time with the higher classification accuracy (AUC of 1.000) as in the ROC analysis. Overall, the AE-PRBFN model is highly accurate and computationally efficient that is much faster than the traditional RBFN model and the SVM model.

## Discussion

The overall statistical analysis shows that the proposed AE-RBFN (Attention Enhanced Phylogenetic Radial Basis Function Network) offers a new level of computational classification of grass species in terms of codon usage patterns and significantly outperforms its ablation approaches as well as known machine learning baselines. The findings shows that there is evident performance pecking order based on architectural innovation. The ideal classification accuracy (1.000) of the full AE-PRBFN model which has been statistically proved via post-hoc tests against all models (p-value < 0.05) proves the synergistic effect of phylogenetic initializing with an attention-enhanced auto-encoder^81,83^. This is a sharp contrast of the Standard RBFN performance, which scored a mere 64.13 per cent accuracy, which is due to its immobile centroids and the inability to optimize the high-dimensional codon features^16,41^. The MLP, which had an almost perfect accuracy (0.9999), and other classical models such as SVM (0.997) and Random Forest (0.995) worked well^27^. But what the AE-PRBFN offers above this accuracy, is an ability to do this, not only with the same level of precision, but in a framework biologically interpretable that explicitly models the constraints of evolution, and is no longer a black-box approach^5,7^.

One of the most important observations of the ablation study is the varying contributions of the main elements of the model. Perhaps this was incidental to this particular well-separated dataset, as AE-RBFN-NoGamma also scored perfectly (1.000) and has no statistically significant difference with the full model (p-value = 0.106). But in marked contrast, the absence of the attention mechanism (AE-PRBFN-NoAttention) led to a major drop in the performance to 81.57% accuracy and this model was statistically worse than the full AE-PRBFN or the NoGamma approach (p-value < 0.05). This definitely determines the attention mechanism as the most critical innovation so that the model is able to learn and prioritize the codon species specific stories which is crucial in separating the closely related species with similar codon usage bias^9,64^. This is further supported by the visualization of attention heat-map (Fig. 5) which shows that each species has specific, biologically conceivable codon preference patterns which agree with known principles of codon usage bias and translational selection^84^.

The AE-PRBFN not only has superior raw accuracy but also superior generalization and training efficiency which practically removes over-fitting. The training and validation accuracy curves meet at zero gap upon completion of few epochs, which is not possible with other models. The MLP, although having high final accuracy, had fluctuations in over-fitting, whereas the Standard RBFN had extreme under-fitting, whereby even increased training could not drive the model to a high-accuracy solution. This is an indication that the integrated auto-encoder does not only offer the feature refinement property but also regularization effect which stabilized the learning process^52,83^. The phylogenetic k-means + + initiation of RBF centroids is an effective type of domain knowledge input by offering a biologically meaningful initiation which, when compared to random initiation, leads to remarkable faster convergence^44,49^. This contributes to the fact that the AE-PRBFN is more accurate, as well as more computationally efficient and reliable to use in practice.

Biological interpretability provided by the model is one of the major steps in the growth of purely discriminative models such as SVM or MLP. The attention weights give a clear exposure of how the model makes its decision-making and the pattern of codon importance that can be assessed against the existing biological knowledge. As an example, the strong emphasis on the AAA (Lysine) codon in a number of species is consistent with its general common use in plants, and the changes in weights of the stop codons (TAA, TGA) could be a clue to lineage-specific differences in genes or translational regulation^2,3^. This ability to derive testable biological propositions based on a classification model overcomes the gap between high-performance machine learning and basic evolutionary genomics^5,6^. It implies possible uses outside classification, including determining the existence of genomic regions that are in a state of unnatural selection, or the design of synthetic genes to produce optimal expression in a target organism^61,88^.

Although these are the strengths, there are some limitations and future directions which need to be noted. The present assessment is limited to four types of grass that belong to Poaceae family. The architecture of the model is generalizable but its accuracy on more distant taxa (e.g. the eudicots) or organisms with vastly different codon usage dynamics (e.g. microbes with high mutation rates) is yet to be evaluated^62,89^. The framework should be checked with the future work on the large phylogenetic levels. In addition, the model needs curated coding sequences, so it might be restricted to non-model organisms with poor annotation^90^. It could be worthwhile to explore semi-supervised or self-supervised methods to take advantage of the large volumes of unidentified genomic data in the effort to overcome this obstacle^7,42^.

## Conclusion

AE-PRBFN framework has proven to be effective in combining the ideas of evolutionary biology, deep learning, and classical theory of neural networks to develop a better tool of genomic classification. It is statistically better than conventional baselines, offers unprecedented training stability, and above all, offers interpretable results on the codon-level features that cause divergence in species. This paper is an example of how much can be gained by integrating domain specific knowledge into neural network architecture and it is progressing towards more understandable and biologically realistic computational models in genomics^19,64^. By expanding the framework to include multi-omics data in the future or by altering it to support real-time and portable applications, it could be expanded even further to use for agricultural biotechnology, conservation, and evolutionary studies.

## Data Availability

The raw sequence data supporting the findings of this study are publicly available from the Ensembl Plants database ([http://plants.ensembl.org/](http:/plants.ensembl.org). The fully processed dataset (codon frequency features and labels) and all codes required to reproduce all results, including data retrieval, filtering, feature extraction, and model fitting are publicly available on GitHub at (https:/github.com/anjumstat/AE_PRBFN_Deep_Learning_Classification.git).

## Abbreviations

AE-PRBFN: Attention enhanced phylogenetic radial basis function networks
RBFN: Radial basis function networks
BD: Brachypodium distachyon
Wheat: Triticum aestivum
Rice: Oryza sativa
Barley: Hordeum vulgare
MCA: Multidimensional cooperative analysis
LDSL: Light weight dual stream learning
ANOVA: Analysis of variance
MLP: Multilayer perceptron
SVM: Support vector machines
CDS: Coding DNA sequences
DNA: Deoxyribonucleic acid
TP: True positives
TN: True negatives
FP: False positives
FN: False negatives
CV: Cross validation
t-SNE: t-distributed stochastic neighbor embedding
UMAP: Uniform manifold approximation and projection
PC: Principal component
PCA: Principal component analysis
RSCU: Relative synonymous codon usage
